# Synthesis and Optical Properties of *N*-Arylnaphtho- and Anthra[2,3-*d*]oxazol-2-amines

**DOI:** 10.3390/molecules30020319

**Published:** 2025-01-15

**Authors:** Yuki Murata, Masato Kawakubo, Ayumi Maruyama, Mio Matsumura, Shuji Yasuike

**Affiliations:** School of Pharmaceutical Sciences, Aichi Gakuin University, 1-100 Kusumoto-cho, Chikusa-ku, Nagoya 464-8650, Japan; y-murata@dpc.agu.ac.jp (Y.M.); ag213a02@dpc.agu.ac.jp (M.K.); yaohuaxue870@gmail.com (A.M.); m-matsu@dpc.agu.ac.jp (M.M.)

**Keywords:** electrochemical reaction, polycyclic benzene-fused oxazole, cyclodesulfurization, thiourea, optical property

## Abstract

Oxazole, a versatile and significant heteroarene, serves as a bridge between synthetic organic chemistry and applications in the medicinal, pharmaceutical, and industrial fields. Polycyclic aromatic compounds with amino groups substituted at the 2-position of an oxazole, such as 2-aminonaphthoxazoles, are expected to be functional probes, but their synthetic methods are extremely limited. Herein, we describe electrochemical reactions of 3-amino-2-naphthol or 3-amino-2-anthracenol and isothiocyanates in DMSO, using a graphite electrode as an anode and a platinum electrode as a cathode in the presence of potassium iodide (KI), which afford *N*-arylnaphtho- and *N*-arylanthra[2,3-*d*]oxazol-2-amines via cyclodesulfurization. This reaction is the first example of synthesis of 2-aminoxazole-based polycyclic compounds using an electrochemical reaction. An examination of the spectroscopic properties of polycyclic oxazoles revealed that the λ_abs_ value of the tetracyclic oxazoles was redshifted relative to that of the tricyclic oxazoles. Moreover, synthesized naphthalene/anthracene-fused tricyclic and tetracyclic oxazoles exhibited extended π-conjugated skeletons and fluoresced in the 340–430 nm region in chloroform.

## 1. Introduction

Oxazoles are important building blocks for the synthesis of functional organic materials and biologically active compounds [1,2,3,4,5]. Among them, *N*-substituted benzo[*d*]oxazol-2-amines (2-aminobenzoxazoles) have garnered considerable attention due to their potential applications in biological and pharmaceutical therapies. For example, suvorexant was recently approved as a medication for treating insomnia [6,7]. Furthermore, 2-aminobenzoxazole derivatives have demonstrated significant inhibitory effects on 5-lipoxygenase and p90 ribosomal S6 kinase, as well as strong α-glucosidase inhibition activity [8,9,10]. As a result, numerous synthetic methods for producing 2-aminobenzoxazoles have been developed [11,12,13]. Moreover, 2-aminonaphthoxazole, which has one more fused benzene ring, has also been reported to be a potential probe for detecting γ-hydroxybutyric acid in soft drinks and alcoholic beverages based on color and fluorescence changes [14]. However, only a few methods are known for the synthesis of its parent 2-aminonaphthoxazole [14,15,16,17,18]. For instance, in 2018, synthesis of *N*-phenylnaphtho[2,3-*d*]oxazol-2-amine via triphenylbismuth dichloride-promoted cyclodesulfurization of thioureas derived from 3-amino-2-naphthol and phenyl isothiocyanate was reported [15]. Since then, Cu_2_O/tetrabutylammonium bromide [16] and elemental sulfur/K_2_CO_3_ systems [17] have been used as reagents for the oxidative cyclodesulfurization reactions, and artificial hemoglobin-containing cobalt porphyrin [18] has been reported to serve as a catalyst in these reactions. However, these studies were more focused on the synthesis of benzoxazoles than on the synthesis of polycyclic *N*-arylnaphtho- and anthra[2,3-*d*]oxazol-2-amines. Moreover, the spectroscopic properties of the synthesized compounds were not investigated despite their potential as functional materials. Electrochemical synthesis attracted attention as an environmentally friendly method [19,20,21]. Bicyclic 2-aminobenzoxazoles have been synthesized through tandem electrochemical reactions from 2-aminophenols and isothiocyanates via thiourea [22]. Inspired by these works, this study aimed to synthesize *N*-aryloxazol-2-amines fused with naphthalene and anthracene rings, namely *N*-arylnaphtho- and *N*-arylanthra[2,3-*d*]oxazol-2-amines, via electrochemical cyclodesulfurization from 3-amino-2-naphthol or 3-amino-2-anthracenol and isothiocyanates. The optical properties of the synthesized compounds were also investigated.

## 2. Results and Discussion

In the electrolytic synthesis of 2-aminobenzoxazoles from 2-aminophenols and isothiocyanates, as reported by Wacharasindhu et al., two reagents, NaI and NaCl, were used as electrolytes and/or reaction mediators with a mixed ethanol–water solvent [22]. Since polycyclic aromatic heterocycles are poorly soluble in water, we attempted to carry out the reaction under simpler electrolysis conditions while considering the solubility of the products. The electrocyclization of thioamide (**3a**) generated from 3-amino-2-naphthol (**1**) and phenyl isothiocyanate (**2a**) in the presence of various electrolytes was carried out to obtain suitable reaction conditions (Table 1). To clarify the effect of the electrolyte, MeCN was used as the solvent with a current of 20 mA using a graphite electrode as the anode [C(+)] and a platinum electrode as the cathode [Pt(−)] (entries 1–5). KI was found to be the best electrolyte for the reaction in terms of the yield of the desired oxazole (**4**). Solvent screening experiments suggested that the reaction proceeded efficiently in DMSO (87%), MeCN (83%), DMF (79%), and DMA (69%) (entries 4 and 6–8), whereas the reaction did not proceed in EtOH or THF (entries 9 and 10). Next, several combinations of the graphite and platinum electrodes were examined. As evident in Table 1, slightly higher yields were obtained when graphite and platinum were used as the anode and cathode, respectively (entries 4 and 11–13). The yield decreased when the KI concentration was reduced (entry 14). When the applied current was reduced from 20 to 5 mA, the reaction time increased (entry 15). Thus, the best result was obtained when **3a**, generated from **1** and **2a**, reacted in DMSO in the presence of a KI (0.5 M) electrolyte at a current density of 20 mA using a graphite [C(+)] anode and a platinum [Pt(−)] cathode (entry 8).

The generality of this protocol was evaluated by examining the reactions of 3-amino-2-naphthol (**1**) or 3-amino-2-anthracenol (**5**) with aryl isothiocyanate (**2**); the results are summarized in Table 2. The reaction of **1** with aryl isothiocyanate (**2**) gave the corresponding tricyclic naphthoxazoles (**6**–**8**) in high yields. When 3-amino-2-anthracenols (**5**) were used as a substrate instead of **1**, the desired tetracyclic anthroxazoles (**9**–**12**) were obtained, albeit in low yields. These lower yields can be attributed to the low solubility of the thiourea intermediates (**3**) derived from **5** and **2**.

**Scheme 1:**
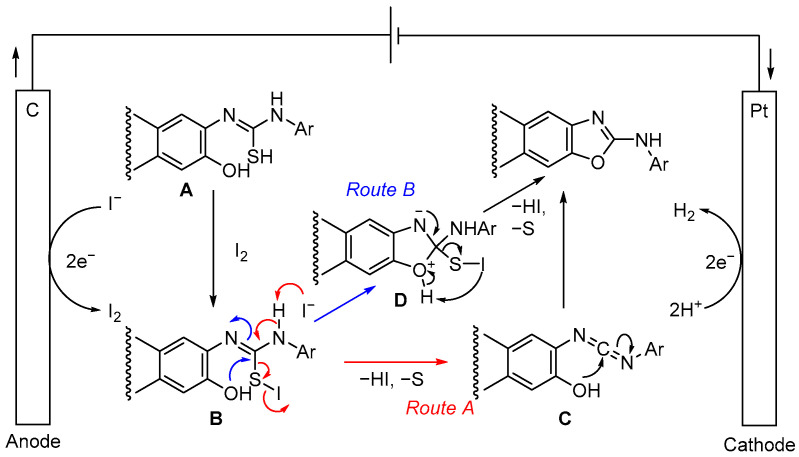
Proposed mechanism.

**Scheme 2:**
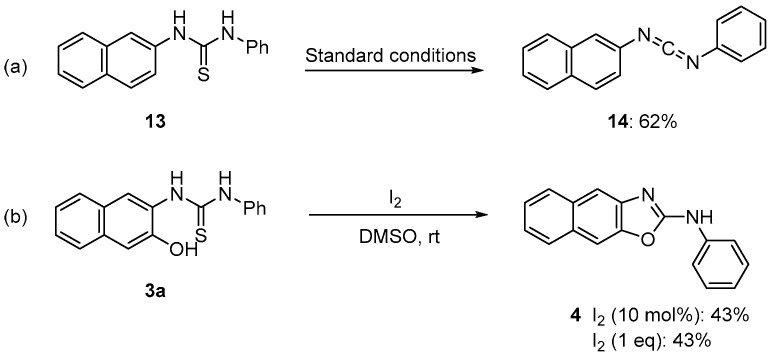
Control reactions. (**a**) Desulfurization of thiourea. (**b**) Experiments using catalytic or equivalent amounts of iodine.

The mechanism of this electrochemical synthesis is unclear at present. However, a plausible mechanism has been proposed (Scheme 1). Iodine (I_2_) is generated from the iodide (I^−^) of an electrolyte such as *n*BuN_4_I, KI, and NaI at the positive electrode of the electrolytic system [23,24,25]. The thiolic form (**A**), a tautomer of thiourea derived from aminophenol and isothiocyanate, reacts with iodine to produce **B**. The electrochemical reaction of 1,3-diphenylthiourea (**13**) under standard conditions affords carbodiimide (**14**) in a 62% yield (Scheme 2a), and the reductive elimination of **B** generates carbodiimide (**C**), sulfur, and hydroiodic acid (Route A). Finally, **C** undergoes intramolecular cyclization to give polycyclic oxazole. Another possible route (Route B) could be the intramolecular cyclization of **B** to give oxazole through dehydrosulfurization via **D**. Reactions of thiourea (**3a**) using catalytic or equivalent amounts of iodine, without electrolysis, gave the corresponding product (**4**) in a 43% yield (Scheme 2b). These results suggest that the addition of iodine alone is less effective and that electrolytic reactions proceed more efficiently using KI, although the reaction mechanism remains unclear.

**Figure 1:**
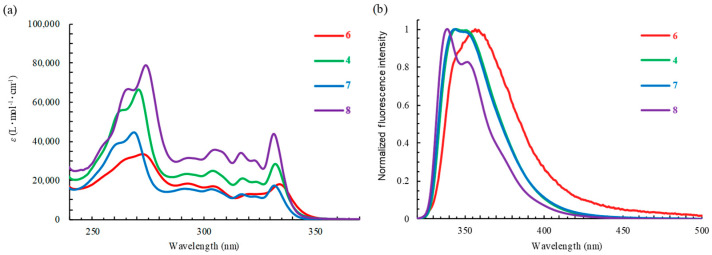
The (**a**) absorption and (**b**) fluorescence spectra of *N*-arylnaphtho[2,3-*d*]oxazol-2-amines **4** and **6**–**8** in CHCl_3_. The excitation wavelength was 310 nm.

**Figure 2:**
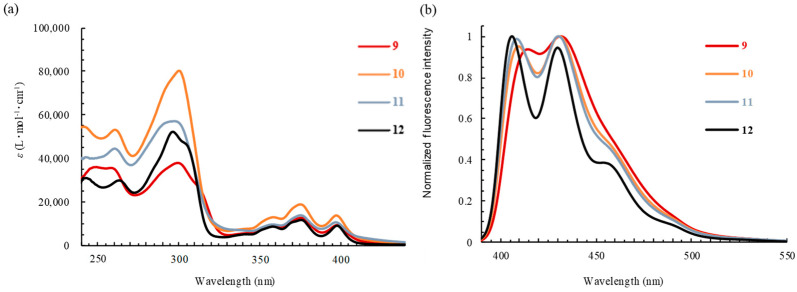
The (**a**) absorption and (**b**) fluorescence spectra of *N*-arylanthra[2,3-*d*]oxazol-2-amines **9**–**12** in CHCl_3_. The excitation wavelength was 380 nm.

The optical properties of tricyclic and tetracyclic oxazole derivatives **4** and **6**–**12** were evaluated based on their UV–Vis absorption and fluorescence spectra in chloroform (Figure 1 and Figure 2 and Table 3). Tricyclic oxazoles **4** and **6**–**8** exhibited an absorption band with a maximum (λ_abs_) at around 330 nm. The absorption maxima were largely unaffected by the substituents at the 4-position of the *N*-phenyl ring. Tetracyclic oxazoles **9**–**12** displayed three bands (λ_abs_) ranging from 350 to 400 nm, which were largely unaffected by the substituents at the 4-position of the *N*-phenyl ring. It is evident that the λ_abs_ value of the tetracyclic oxazoles was redshifted by ~70 nm relative to that of the tricyclic oxazoles, as presented in Table 3. Compounds **4** and **6**–**8** exhibited fluorescence maxima (λ_em_) at 339–357 nm. The 4-methoxyphenyl derivative (**6**) exhibited negligible fluorescence, while the 4-trifluoromethyl derivative (**8**) showed stronger fluorescence (*Φ*_F_ = 32%) than compounds **4** and **7**. Furthermore, the λ_em_ of **4** and **6**–**8** was redshifted from 339 to 357 nm with an increase in the electron-donating ability of the substituents at the 4-position on the *N*-phenyl ring. Tetracyclic oxazoles **9**–**12** exhibited two fluorescence emission bands at around 410 and 430 nm, with *Φ*_F_ values of 14–27%. The λ_em_ values of tetracyclic oxazoles **9**–**12** were not affected by the electronic property of the substituent on the *N*-phenyl ring.

## 3. Materials and Methods

### 3.1. General Information

Unless otherwise stated, all reagents and solvents were purchased from commercial suppliers and used without further purification. ^1^H NMR (DMSO: *δ* 2.48 ppm as an internal standard), ^13^C NMR (DMSO-*d*_6_: *δ* 39.5 ppm as an internal standard), and ^19^F NMR (trifluoromethylbenzene: *δ* −64.0 ppm as an external standard) spectra were recorded on a JEOL ECZ-400S (400 MHz, 100 MHz, and 376 MHz) spectrometer (JEOL Ltd., Tokyo, Japan) in DMSO-*d*_6_. Melting points were measured on a Yanagimoto micro melting point hot stage apparatus (Yanaco Technical Science Co., Ltd., Tokyo, Japan) and were not corrected. GC-MS (EI) spectra were recorded on an Agilent 5977E Diff-SST MSD-230V spectrometer (Agilent Technologies Japan, Ltd., Tokyo, Japan). HRMS (ESI) were measured on an Agilent 6230 TOF mass spectrometer (Agilent Technologies Japan, Ltd., Tokyo, Japan). IR spectra were recorded on an FTIR-8400S system from Shimadzu (SHIMADZU Corp., Kyoto, Japan) and are reported using the frequency of absorption (cm^−1^). UV–Vis spectra were recorded at room temperature on a HITACHI U-2800A spectrophotometer (Hitachi High-Tech Corporation, Tokyo, Japan, C = 1.30 × 10^−5^–2.98 × 10^−5^ M in CHCl_3_), and fluorescence spectra were recorded on a JASCO FP-8300 luminescence spectrometer (JASCO Corporation, Tokyo, Japan, C = 1.51 × 10^−6^–4.73 × 10^−6^ M in CHCl_3_). Only selected IR absorbencies are reported. All chromatographic separations were accomplished with Silica Gel 60N (Kanto Chemical Co., Inc., Tokyo, Japan). Thin-layer chromatography (TLC) was performed with Macherey-Nagel Sil G25 UV254 pre-coated TLC plates. 3-Amino-2-naphthol (**1**) and isothiocyanates (**2**) were purchased from TCI Fine Chemicals, Japan. Aminophenol (**5**) [27] was prepared according to the reported procedure. All electrochemical reactions were carried out in an IKA ElectraSyn 2.0 using ElectraSyn 2.0 undivided cells (a 5 mL vial) equipped with standard IKA ElectraSyn 2.0 electrodes (IKA Japan K.K., Osaka, Japan).

### 3.2. Synthesis of N-Arylnaphtho- and Anthra[2,3-d]oxazol-2-amines

In a 5.0 mL ElectraSyn vial, a mixture of aminophenol (**1** or **5**: 0.4 mmol) and arylisothiocyanate (**2**: 0.4 mmol) in DMSO (5.0 mL) was stirred well for 18 h. After complete conversion of the reaction, KI (83.0 mg, 0.5 mmol, and 0.1 M) was added to the reaction mixture. The vial was sealed with an ElectraSyn vial cap fitted with graphite SK-50 as an anode and platinum foil as a cathode. Constant-current electrolysis was performed at 20 mA for 2–4 h. The reaction mixture was diluted with H_2_O (20 mL) and AcOEt (20 mL), and the aqueous phase was extracted with AcOEt (3 × 30 mL). The combined organic phase was washed with brine (20 mL) and dried over MgSO_4_. Evaporation of the solvent provided the crude product. The crude product was then purified by column chromatography on silica gel (copies of ^1^H and ^13^C NMR see Supplementary Materials).

### 3.3. Characterization Data

#### 3.3.1. *N*-Phenylnaphtho[2,3-*d*]oxazol-2-amine (**4**) [15]

Yellow prisms (90.4 mg, 87%), m.p.: 198–200 °C (from CH_2_Cl_2_-Hexane), *R*_f_ = 0.45 (Hexane/AcOEt = 8:2). ^1^H NMR (400 MHz, DMSO-*d*_6_): *δ* 10.9 (brs, 1H, NH), 7.93–7.90 (m, 3H, Ar-H), 7.84 (s, 1H, Ar-H), 7.79 (d, *J* = 8.0 Hz, 2H, Ar-H), 7.42–7.37 (m, 4H, Ar-H), and 7.05 (t, *J* = 7.6 Hz, 1H, Ar-H) ppm. ^13^C NMR (100 MHz, DMSO-*d*_6_): *δ* 159.3 (C), 146.9 (C), 142.9 (C), 138.4 (C), 131.3 (C), 129.6 (C), 129.0 (CH), 127.6 (CH), 127.5 (CH), 124.4 (CH), 124.0 (CH), 122.6 (CH), 118.0 (CH), 112.3 (CH), and 104.5 (CH) ppm. HRMS: *m*/*z* [M + H]^+^ calculated for C_17_H_13_N_2_O: 261.1022. Found: 261.1019. IR (KBr): *ν* 3019 (NH) cm^−1^.

#### 3.3.2. *N*-(4-Methoxyphenyl)naphtho[2,3-*d*]oxazol-2-amine (**6**) [16]

Colorless needles (104 mg, 90%), m.p.: 190–191 °C (from CH_2_Cl_2_), *R*_f_ = 0.40 (Hexane/AcOEt = 8:2). ^1^H NMR (400 MHz, DMSO-*d*_6_): *δ* 10.6 (brs, 1H, NH), 7.92–7.88 (m, 3H, Ar-H), 7.78 (s, 1H, Ar-H), 7.68 (td, *J* = 9.2, 2.9 Hz, 2H, Ar-H), 7.41–7.35 (m, 2H, Ar-H), 6.96 (td, *J* = 9.2, 2.9 Hz, 2H, Ar-H), and 3.73 (s, 3H, OCH_3_) ppm. ^13^C NMR (100 MHz, DMSO-*d*_6_): *δ* 160.2 (C), 155.5 (C), 147.7 (C), 143.7 (C), 132.1 (C), 131.9 (C), 130.0 (C), 128.2 (CH), 128.0 (CH), 124.9 (CH), 124.5 (CH), 120.2 (CH), 114.9 (CH), 112.5 (CH), 104.9 (CH), and 55.9 (CH_3_) ppm. HRMS: *m*/*z* [M + H]^+^ calculated for C_18_H_15_N_2_O_2_: 291.1128. Found: 291.1118. IR (KBr): *ν* 3018 (NH) cm^−1^.

#### 3.3.3. *N*-(4-Fluorophenyl)naphtho[2,3-*d*]oxazol-2-amine (**7**)

Colorless prisms (95.6 mg, 86%), m.p.: 250–252 °C (from CH_2_Cl_2_-Hexane), *R*_f_ = 0.4 (Hexane/AcOEt = 9:1). ^1^H NMR (400 MHz, DMSO-*d*_6_): *δ* 10.9 (brs, 1H, NH), 7.93–7.89 (m, 3H, Ar-H), 7.82–7.78 (m, 3H, Ar-H), 7.42–7.36 (m, 2H, Ar-H), and 7.23 (tt, *J* = 9.2, 3.6 Hz, 2H, Ar-H) ppm. ^13^C NMR (100 MHz, DMSO-*d*_6_): *δ* 159.9 (C), 158.3 (d, ^1^*J*_C,F_ = 237 Hz, C), 147.6 (C), 143.4 (C), 135.4 (C), 131.9 (C), 130.1 (C), 128.2 (CH), 128.0 (CH), 125.0 (CH), 124.6 (CH), 120.2 (d, ^3^*J*_C,F_ = 7.7 Hz, CH), 116.2 (d, ^2^*J*_C,F_ = 22.0 Hz, CH), 112.9 (CH), and 105.1 (CH) ppm. ^19^F NMR (376 MHz, DMSO-*d*_6_): *δ* −120.4 ppm. HRMS: *m*/*z* [M + H]^+^ calculated for C_17_H_12_FN_2_O: 279.0928. Found: 279.0923. IR (KBr): *ν* 3057 (NH) cm^−1^.

#### 3.3.4. *N*-[4-(Trifluoromethyl)phenyl]naphtho[2,3-*d*]oxazol-2-amine (**8**)

Colorless prisms (116 mg, 89%), m.p.: 201–202 °C (from CH_2_Cl_2_-Hexane), *R*_f_ = 0.43 (Hexane/AcOEt = 8:2). ^1^H NMR (400 MHz, DMSO-*d*_6_): *δ* 11.3 (brs, 1H, NH), 8.00–7.90 (m, 6H, Ar-H), 7.76 (d, *J* = 9.2 Hz, 2H, Ar-H), and 7.45–7.39 (m, 2H, Ar-H) ppm. ^13^C NMR (100 MHz, DMSO-*d*_6_): *δ* 159.3 (C), 147.4 (C), 142.9 (C), 142.6 (C), 131.8 (C), 130.4 (C), 128.3 (CH), 128.2 (CH), 127.0 (q, ^3^*J*_C,F_ = 3.8 Hz, CH), 125.4 (q, ^1^*J*_C,F_ = 270 Hz, C), 125.1 (CH), 124.9 (CH), 123.0 (q, ^2^*J*_C,F_ = 31.6 Hz, C), 118.4 (CH), 113.5 (CH), and 105.4 (CH) ppm. ^19^F NMR (376 MHz, DMSO-*d*_6_): *δ* −60.0 ppm. HRMS: *m*/*z* [M + H]^+^ calculated for C_18_H_12_F_3_N_2_O: 329.0896. Found: 329.0888. IR (KBr): *ν* 3018 (NH) cm^−1^.

#### 3.3.5. *N*-(4-Methoxyphenyl)anthra[2,3-*d*]oxazol-2-amine (**9**)

Yellow powder (23.1 mg, 17%), m.p. > 300 °C (from THF-Hexane), *R*_f_ = 0.40 (Hexane/AcOEt = 7:3). ^1^H NMR (400 MHz, DMSO-*d*_6_): *δ* 10.8 (brs, 1H, NH), 8.52 (d, *J* = 7.9 Hz, 2H, Ar-H), 7.99 (t, *J* = 4.6 Hz, 3H, Ar-H), 7.88 (s, 1H, Ar-H), 7.69 (d, *J* = 8.6 Hz, 2H, Ar-H), 7.43–7.39 (m, 2H, Ar-H), 6.98 (d, *J* = 9.1 Hz, 2H, Ar-H), and 3.74 (s, 3H, OCH_3_) ppm. ^13^C NMR (100 MHz, DMSO-*d*_6_): *δ* 160.5 (C), 155.7 (C), 148.3 (C), 144.6 (C), 137.9 (C), 131.8 (C), 131.7 (C), 130.9 (CH), 130.5 (C), 129.2 (C), 128.14 (CH), 128.05 (CH), 126.1 (CH), 125.5 (CH), 125.3 (CH), 120.4 (CH), 114.9 (CH), 111.2 (CH), 103.8 (CH), and 55.9 (CH_3_) ppm. HRMS: *m*/*z* [M + H]^+^ calculated for C_22_H_17_N_2_O_2_: 341.1285. Found: 341.1275. IR (KBr): *ν* 2955 (NH) cm^−1^.

#### 3.3.6. *N*-Phenylanthra[2,3-*d*]oxazol-2-amine (**10**) [15]

Yellow powder (44.7 mg, 36%), m.p.: 278–281 °C (from AcOEt-Hexane), *R_f_* = 0.37 (Hexane/AcOEt = 8:2). ^1^H NMR (400 MHz, DMSO-*d*_6_): *δ* 10.9 (brs, 1H, NH), 8.56 (d, *J* = 4.4 Hz, 2H, Ar-H), 8.03–8.00 (m, 3H, Ar-H), 7.95 (s, 1H, Ar-H), 7.81 (d, *J* = 7.2 Hz, 2H, Ar-H), 7.45–7.38 (m, 4H, Ar-H), and 7.08 (t, *J* = 7.6 Hz, 1H, Ar-H) ppm. ^13^C NMR (100 MHz, DMSO-*d*_6_): *δ* 159.7 (C), 147.5 (C), 143.7 (C), 138.2 (C), 130.3 (C), 130.2 (C), 129.9 (C), 129.0 (CH), 128.7 (CH), 127.6 (CH), 127.5 (CH), 125.5 (CH), 125.1 (CH), 124.9 (CH), 124.7 (CH), 122.8 (CH), 118.2 (CH), 111.0 (CH), and 103.3 (CH) ppm. HRMS: *m*/*z* [M + H]^+^ calculated for C_21_H_15_N_2_O: 311.1179. Found: 311.1168. IR (KBr): *ν* 2997 (NH) cm^−1^.

#### 3.3.7. *N*-(4-Fluorophenyl)anthra[2,3-*d*]oxazol-2-amine (**11**)

Yellow prisms (61.7 mg, 47%), m.p.: 285–286 °C (from AcOEt-Hexane), *R*_f_ = 0.25 (Hexane/AcOEt = 8:2). ^1^H NMR (400 MHz, DMSO-*d*_6_): *δ* 11.0 (brs, 1H, NH), 8.55 (d, *J* = 4.8 Hz, 2H, Ar-H), 8.03–8.00 (m, 3H, Ar-H), 7.94 (s, 1H, Ar-H), 7.84–7.81 (m, 2H, Ar-H), 7.46–7.41 (m, 2H, Ar-H), and 7.26 (t, *J* = 8.0 Hz, Ar-H) ppm. ^13^C NMR (100 MHz, DMSO-*d*_6_): *δ* 160.3 (C), 158.4 (d, ^1^*J*_C,F_ = 237 Hz, C), 148.1 (C), 144.2 (C), 135.2 (C), 130.9 (C), 130.8 (C), 130.5 (C), 129.2 (C), 128.13 (CH), 128.11 (CH), 126.1 (C), 125.7 (C), 125.5 (CH), 125.3 (CH), 120.3 (d, ^3^*J*_C,F_ = 7.7 Hz, CH), 116.3 (d, ^2^*J*_C,F_ = 23.0 Hz, CH), 111.6 (CH), and 103.9 (CH) ppm. ^19^F NMR (376 MHz, DMSO-*d*_6_): *δ* -120.1 ppm. HRMS: *m*/*z* [M^+^] calculated for C_21_H_14_FN_2_O: 329.1085. Found: 329.1076. IR (KBr): *ν* 2928 (NH) cm^−1^.

#### 3.3.8. *N*-[4-(Trifluoromethyl)phenyl]anthra[2,3-*d*]oxazol-2-amine (**12**)

Yellow powder (80.1 mg, 53%), m.p. > 300 °C (from THF-Hexane), *R*_f_ = 0.35 (Hexane/AcOEt = 8:2). ^1^H NMR (400 MHz, DMSO-*d*_6_): *δ* 11.4 (brs, 1H, NH), 8.57 (s, 2H, Ar-H), 8.08 (s, 1H, Ar-H), 8.03–8.00 (m, 5H, Ar-H), 7.77 (d, *J* = 8.2 Hz, 2H, Ar-H), and 7.44–7.42 (m, 2H, Ar-H) ppm. ^13^C NMR (100 MHz, DMSO-*d*_6_): *δ* 159.8 (C), 147.9 (C), 143.8 (C), 142.4 (C), 130.9 (C), 130.73 (C), 130.69 (C), 129.4 (C), 128.1 (q, ^3^*J*_C,F_ = 4.8 Hz, CH), 127.0 (q, ^4^*J*_C,F_ = 3.8 Hz, CH), 126.2 (CH), 125.9 (CH), 125.7 (CH), 125.5 (CH), 124.9 (q, ^1^*J*_C,F_ = 273 Hz, C), 123.3 (q, ^2^*J*_C,F_ = 31.3 Hz, C), 118.7 × 2 (CH), 112.4 (CH), and 104.3 (CH) ppm. ^19^F NMR (376 MHz, DMSO-*d*_6_): *δ* −60.0 ppm. HRMS: *m*/*z* [M + H]^+^ calculated for C_22_H_14_F_3_N_2_O: 379.1053. Found: 379.1047. IR (KBr): *ν* 3059 (NH) cm^−1^.

## 4. Conclusions

In conclusion, we have demonstrated a simple method for the synthesis of polycyclic benzene-fused oxazoles using electrochemical cyclodesulfurization reactions. These are environmentally friendly reactions that use commercially available and inexpensive KI as an electrolytic mediator. Oxazoles **4** and **6**–**12** exhibited fluorescence in the 340–430 nm region in chloroform. Detailed mechanistic investigations of these electrochemical reactions, structural characterization, and elucidation of the physical properties of the polycyclic oxazoles are currently underway in our laboratory.

## Figures and Tables

**Table 1:** Screening of reaction conditions ^a^.

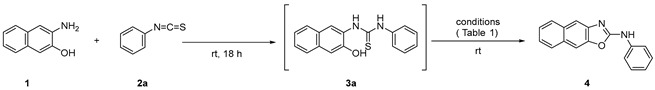						
**Entry**	**Electrolyte**	**Electrode**	**Current (mA)**	**Solvent**	**Time (h)**	**Yield (%) ^b^**
1	NaI	C(+)/Pt(−)	20	MeCN	3	64
2	NH_4_I	C(+)/Pt(−)	20	MeCN	3	77
3	*n*Bu_4_NI	C(+)/Pt(−)	20	MeCN	4	76
4	KI	C(+)/Pt(−)	20	MeCN	3	83
5	KBr	C(+)/Pt(−)	20	MeCN	24	---
6	KI	C(+)/Pt(−)	20	DMF	3	79
7	KI	C(+)/Pt(−)	20	DMA	3	69
8	KI	C(+)/Pt(−)	20	DMSO	2	87
9	KI	C(+)/Pt(−)	20	EtOH	24	---
10	KI	C(+)/Pt(−)	20	THF	24	---
11	KI	C(+)/C(–)	20	DMSO	2	72
12	KI	Pt(+)/Pt(−)	20	DMSO	3	81
13	KI	Pt(+)/C(–)	20	DMSO	4	81
14 ^c^	KI	C(+)/Pt(−)	20	DMSO	3	60
15	KI	C(+)/Pt(−)	5	DMSO	24	80

^a^ **1a** (0.4 mmol), **2a** (0.4 mmol), electrolyte (0.5 mmol, 0.1 M), and solvent (5 mL). ^b^ Yield of isolated product. ^c^ Electrolyte (0.024 M).

**Table 2:** Synthesis of polycyclic *N*-aryloxazol-2-amines ^a,b^.

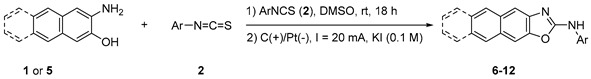					
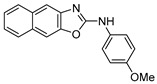	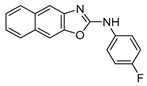		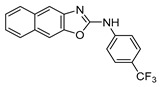		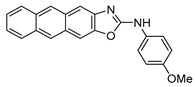
**6**: 90% (4 h)	**7**: 86% (2 h)		**8**: 89% (2 h)		**9**: 17% (4 h)
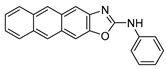		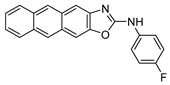		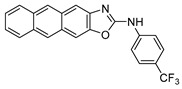	
**10**: 36% (2 h)		**11**: 47% (2 h)		**12**: 53% (2 h)	

^a^ All reactions were carried out using **1** or **5** (0.4 mmol), **2** (0.4 mmol), KI (0.5 mmol, 0.1 M), and DMSO (5 mL). ^b^ Yield of isolated product.

**Table 3:** Absorption (C = 1.30 × 10^−5^–2.98 × 10^−5^ M) and fluorescence (C = 1.51 × 10^−6^–4.73 × 10^−6^ M) data for **4** and **6**–**12** in CHCl_3_ at room temperature.

Compound	Ar	λ_abs_ (nm)	λ_em_ (nm) ^a^	*Φ*_F_ (%) ^a,b^
**6**	4-MeOC_6_H_4_	334	357	<1
**4**	C_6_H_5_	332	345	15
**7**	4-FC_6_H_4_	332	344	11
**8**	4-CF_3_C_6_H_4_	332	339	32
**9**	4-MeOC_6_H_4_	359, 376, 398	414, 431	27
**10**	C_6_H_5_	359, 376, 398	409, 431	18
**11**	4-FC_6_H_4_	359, 376, 398	408, 430	14
**12**	4-CF_3_C_6_H_4_	359, 376, 398	406, 430	20

^a^ Excitation at **310** (**4** and **6**–**8**) or **380** (**9**–**12**) nm. ^b^ The relative quantum yields were estimated using a solution of anthracene in ethanol (*Φ*_F_ = 0.27) for **4** and **6**–**8** and using 9,10-diphenyl anthracene in cyclohexane (*Φ*_F_ = 0.90) for **9**–**12** [26].

## Data Availability

Data are available from the authors upon reasonable request.

## Supplementary Materials

The following supporting information can be downloaded at https://www.mdpi.com/article/10.3390/molecules30020319/s1.
