# Supplementary material for: Synthesis and Optical Properties of N-Arylnaphtho- and Anthra[2,3-d]oxazol-2-amines
Source: Molecules. 2025 Jan 15;30(2):319. doi: 10.3390/molecules30020319 (PMC11767873; doi:10.3390/molecules30020319)

Supporting Information
for
Synthesis and optical properties of *N*-arylnaphtho- and anthra[2,3-*d*]oxazol-2-amines

Yuki Murata, Masato Kawakubo, Ayumi Maruyama, Mio Matsumura, and Shuji Yasuike *

School of Pharmaceutical Sciences, Aichi Gakuin University, 1-100 Kusumoto-cho, Chikusa-ku, Nagoya 464-8650, Japan

E-mail: s-yasuik@dpc.agu.ac.jp

*Corresponding author.

Table of contents

1. Copies of ^{1}H, ^{13}C and ^{19}F NMR spectra S1

1. Copies of ^{1}H, ^{13}C and ^{19}F NMR spectra

^{1}H NMR of **4**

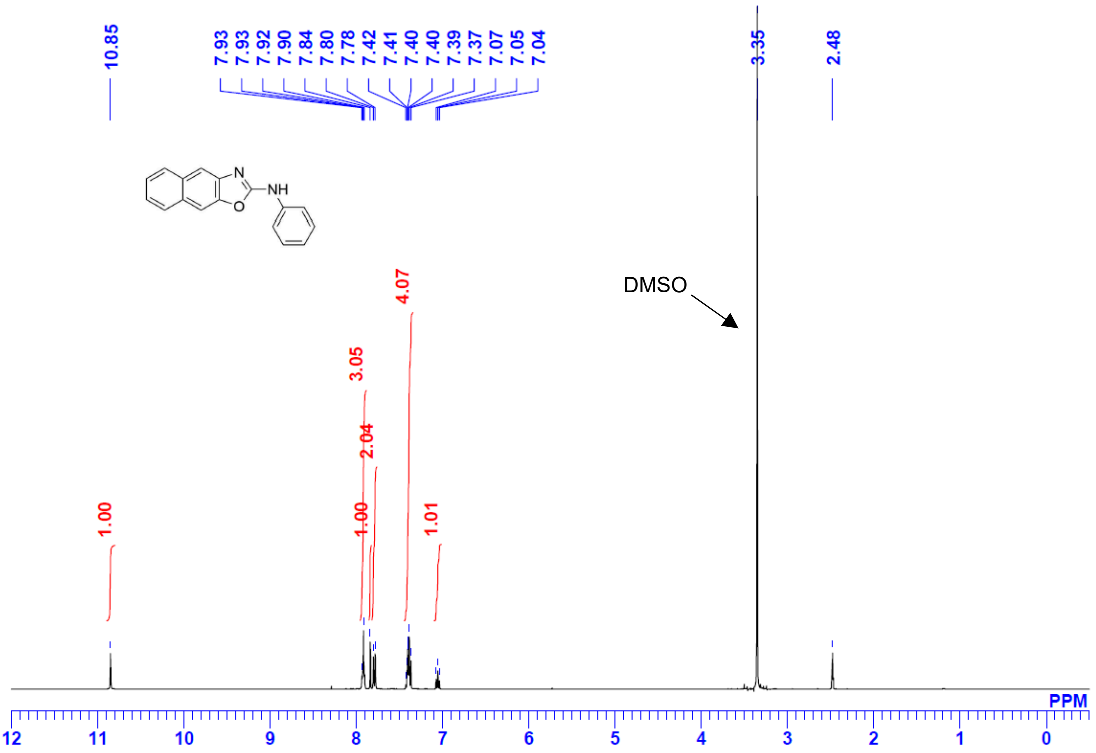

^{13}C NMR of **4**

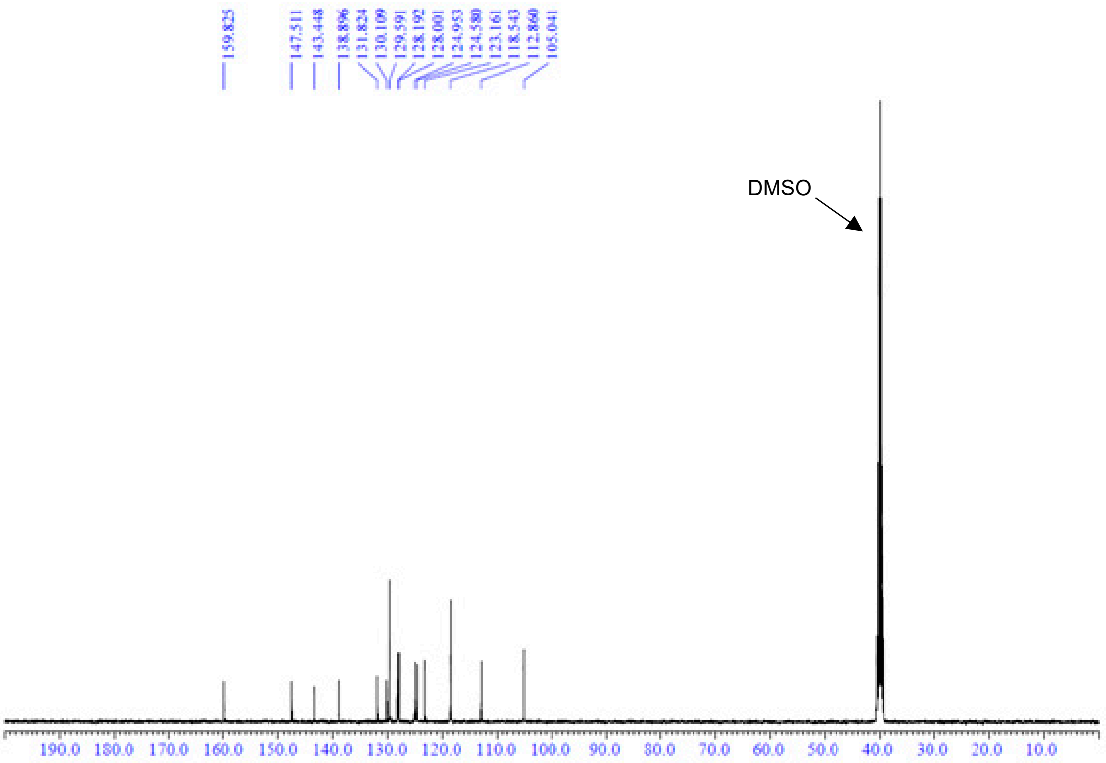

^1H NMR of **6**

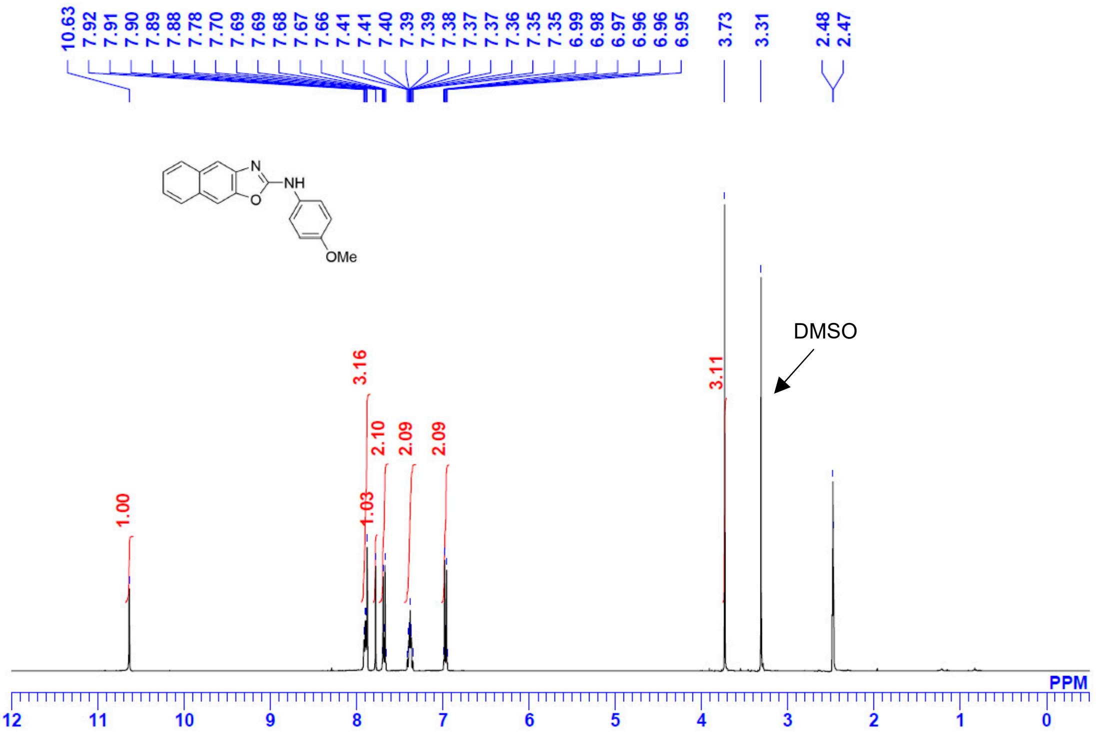

^{13}C NMR of **6**

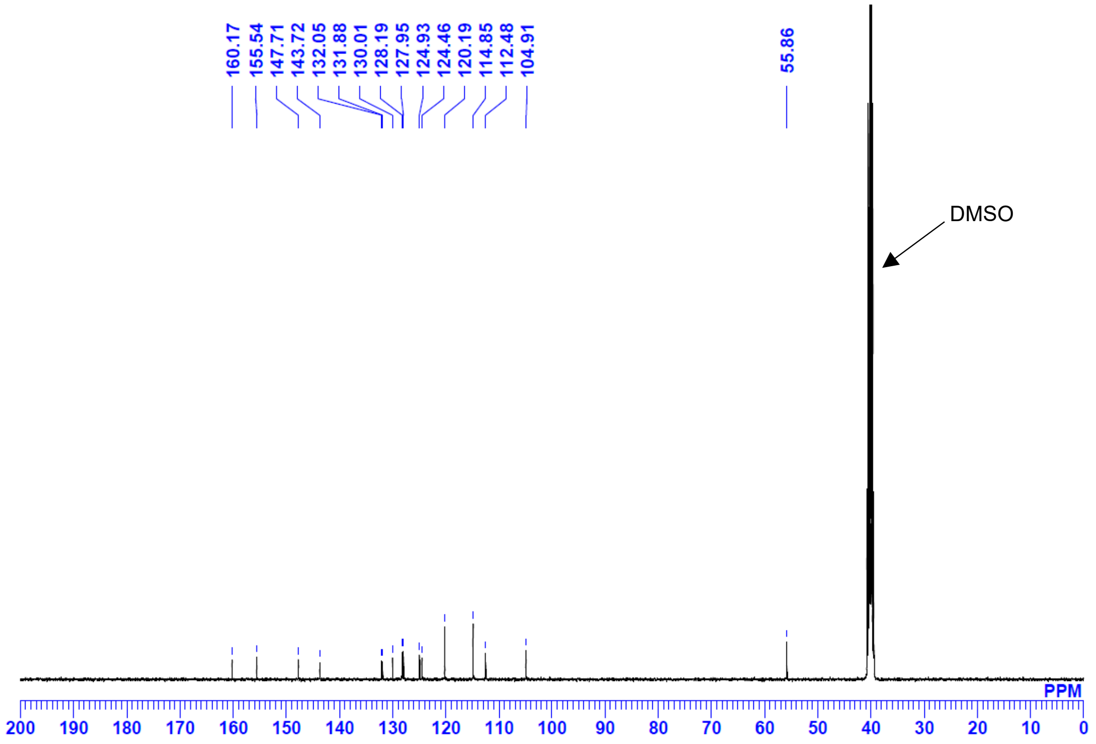

^{1}H NMR of **7**

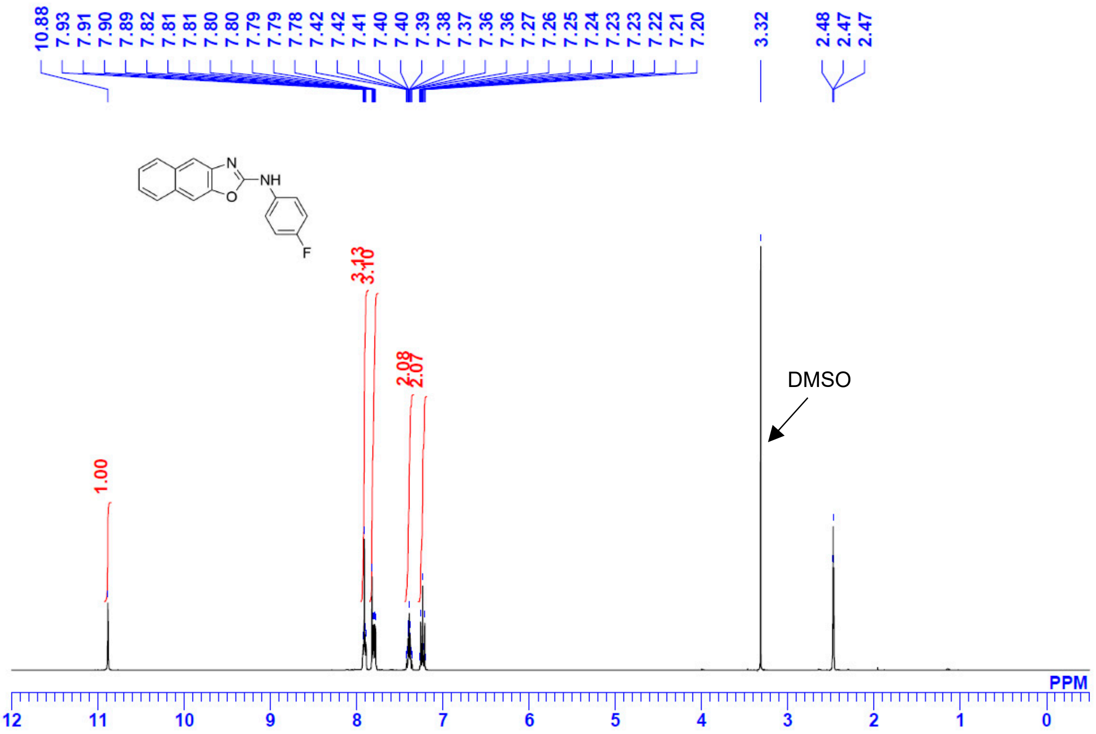

^{13}C NMR of **7**

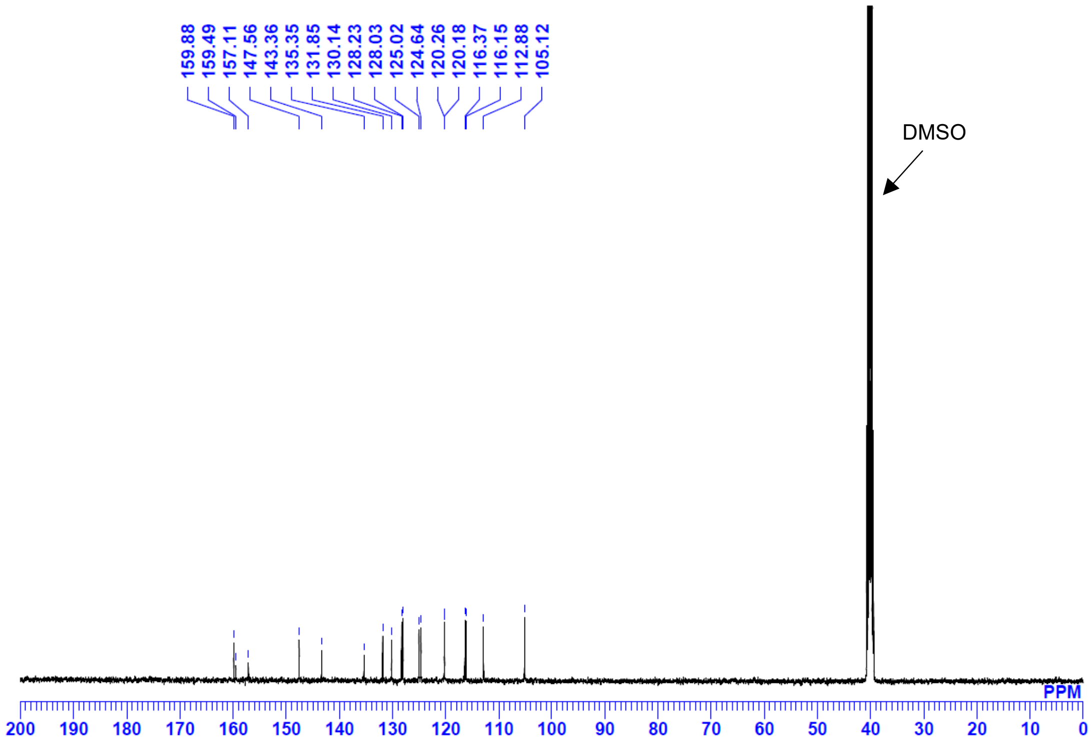

^{19}F NMR of **7**

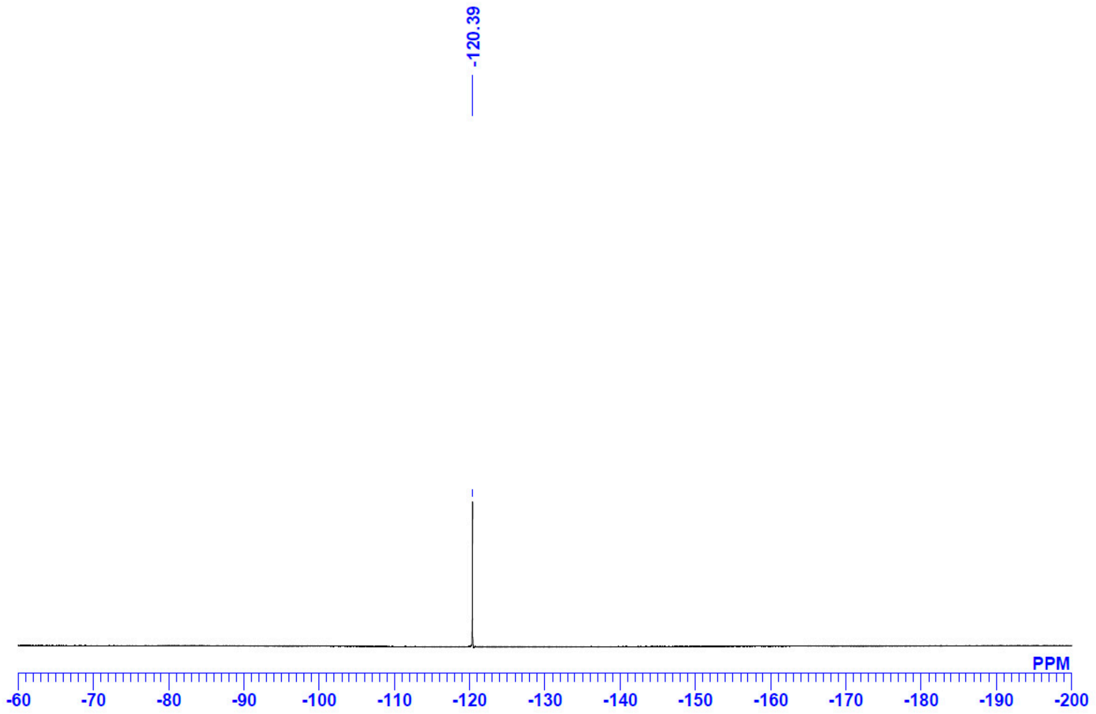

^{1}H NMR of **8**

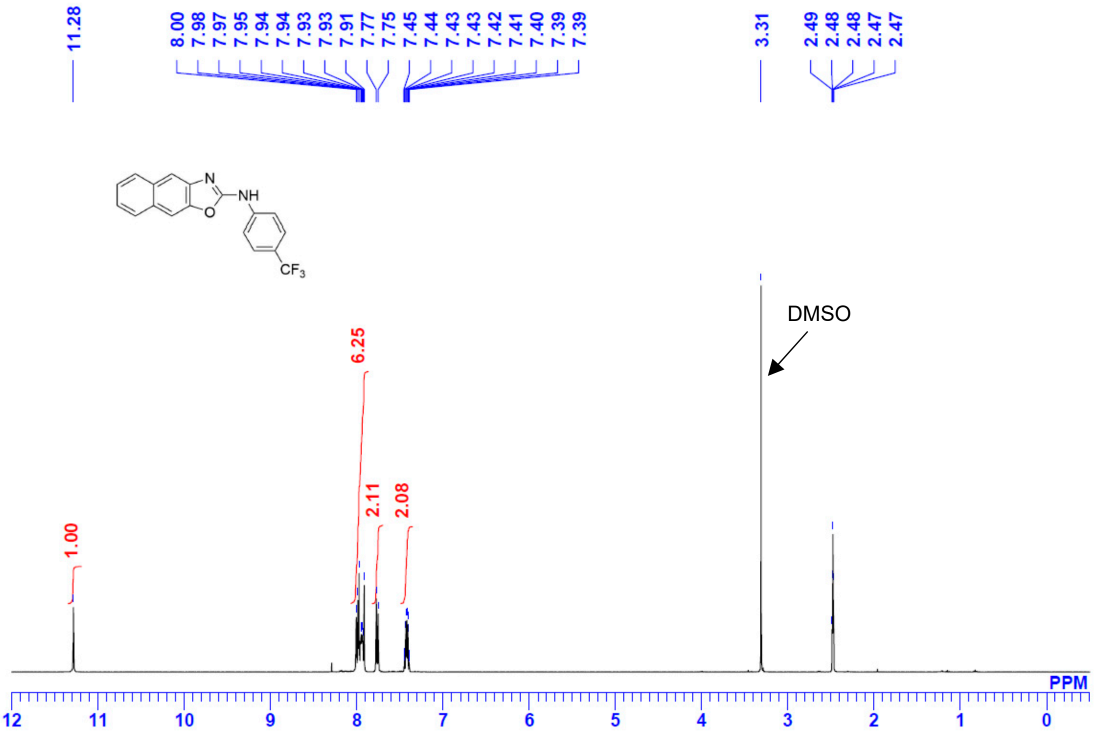

^{13}C NMR of **8**

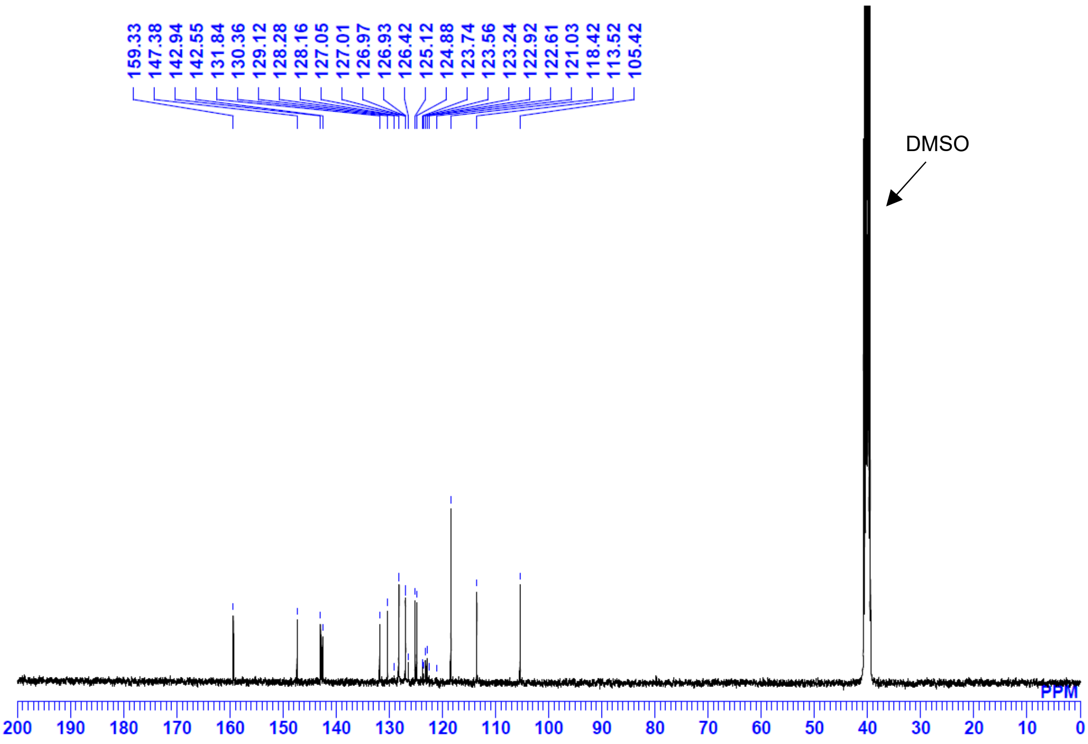

^{19}F NMR of **8**

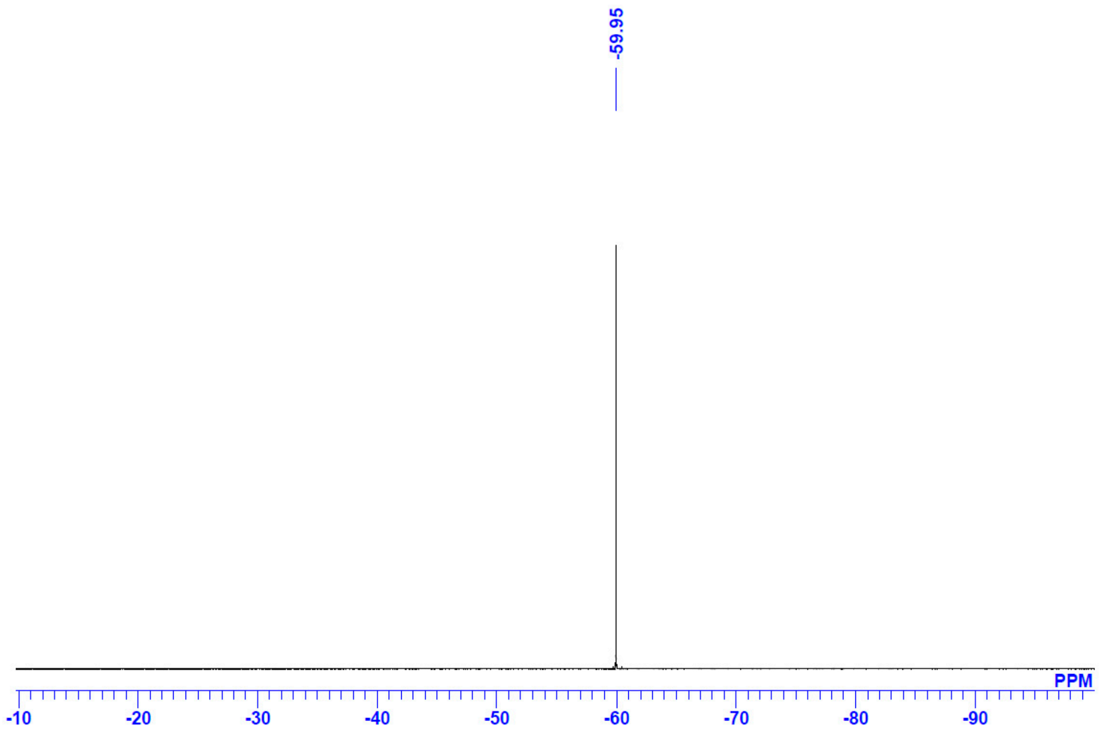

^{1}H NMR of **9**

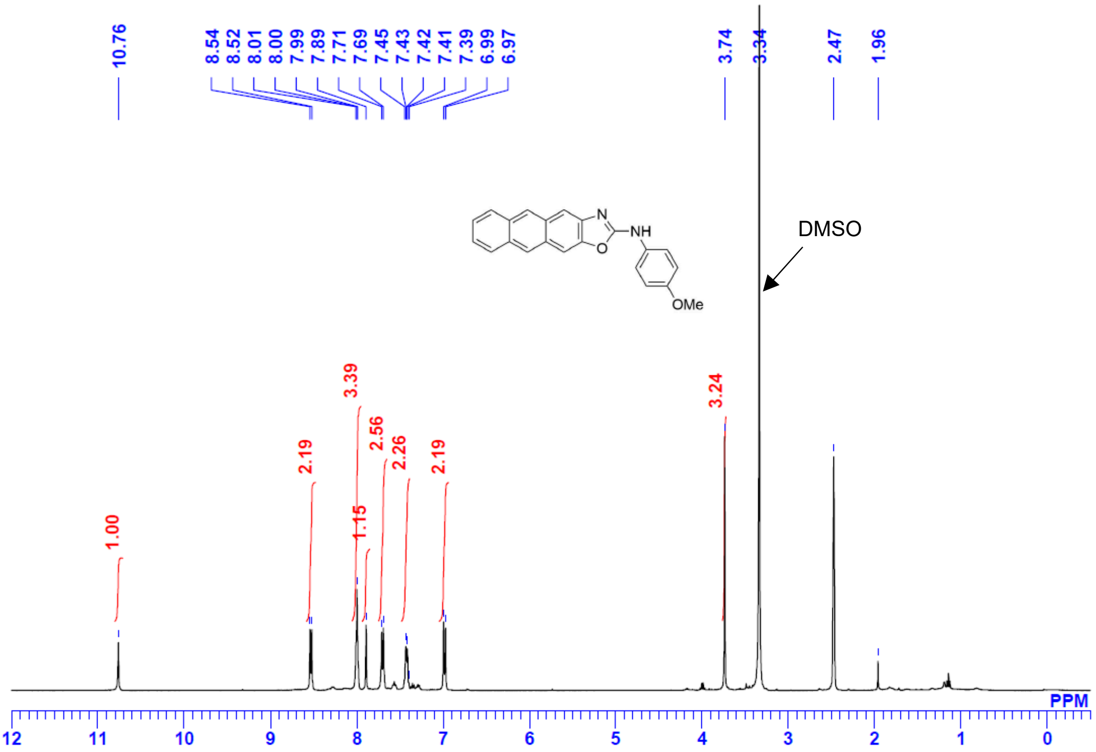

^{13}C NMR of **9**

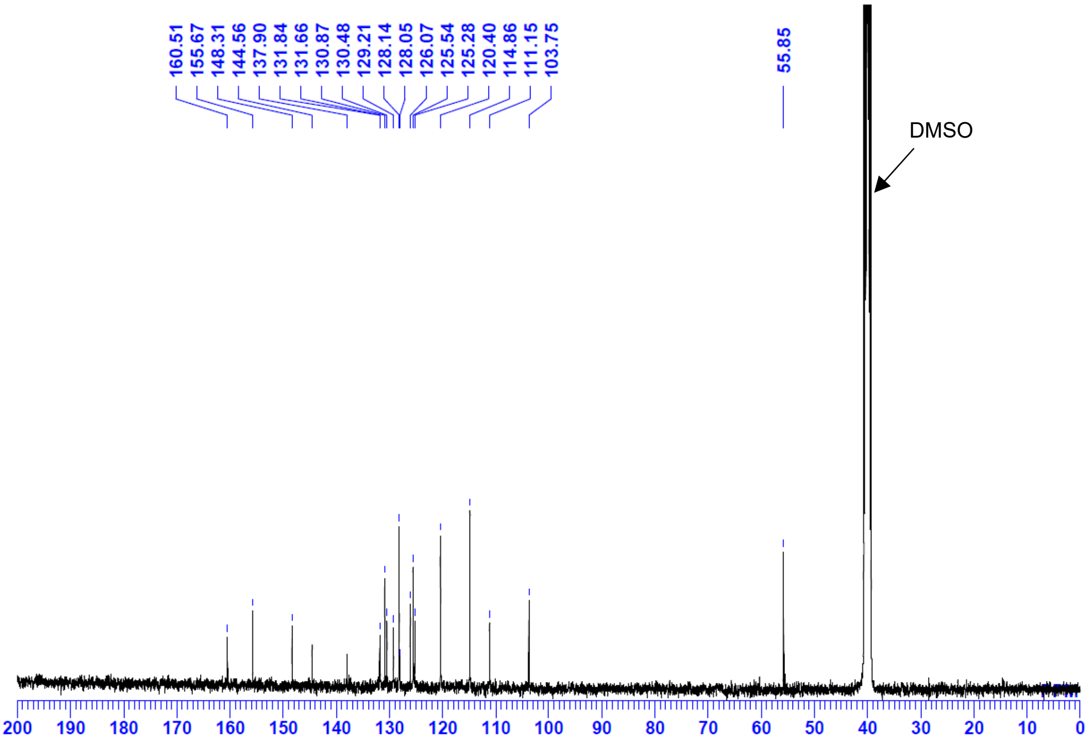

^{1}H NMR of **10**

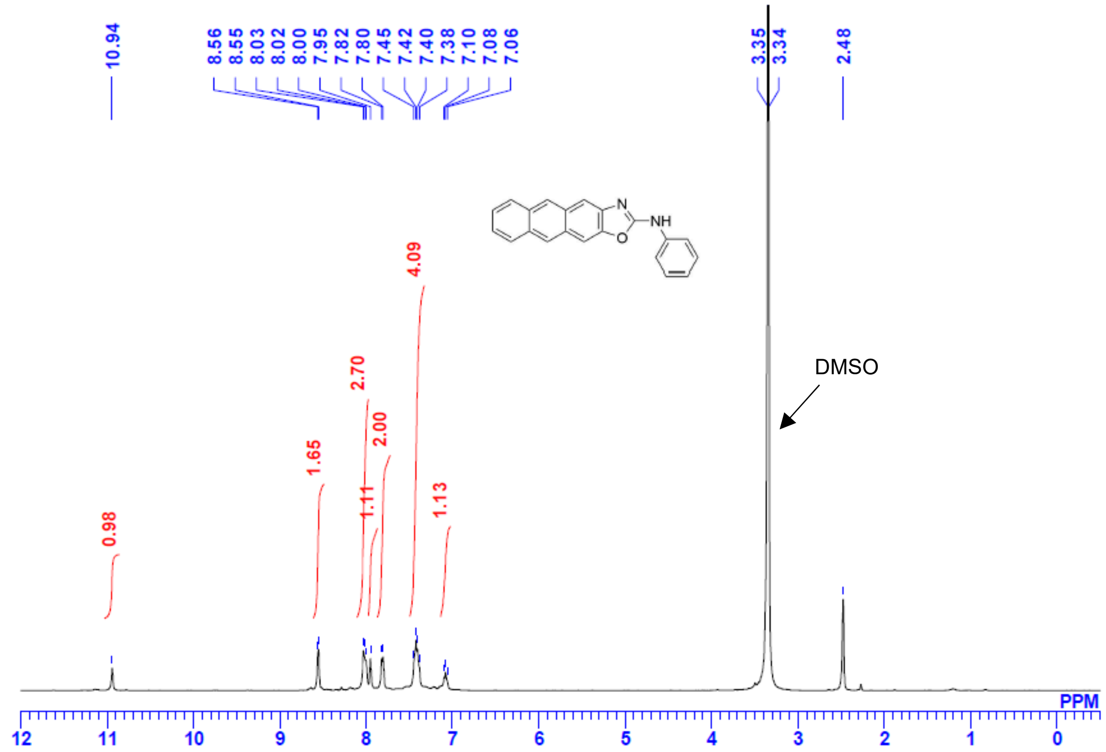

^{13}C NMR of **10**

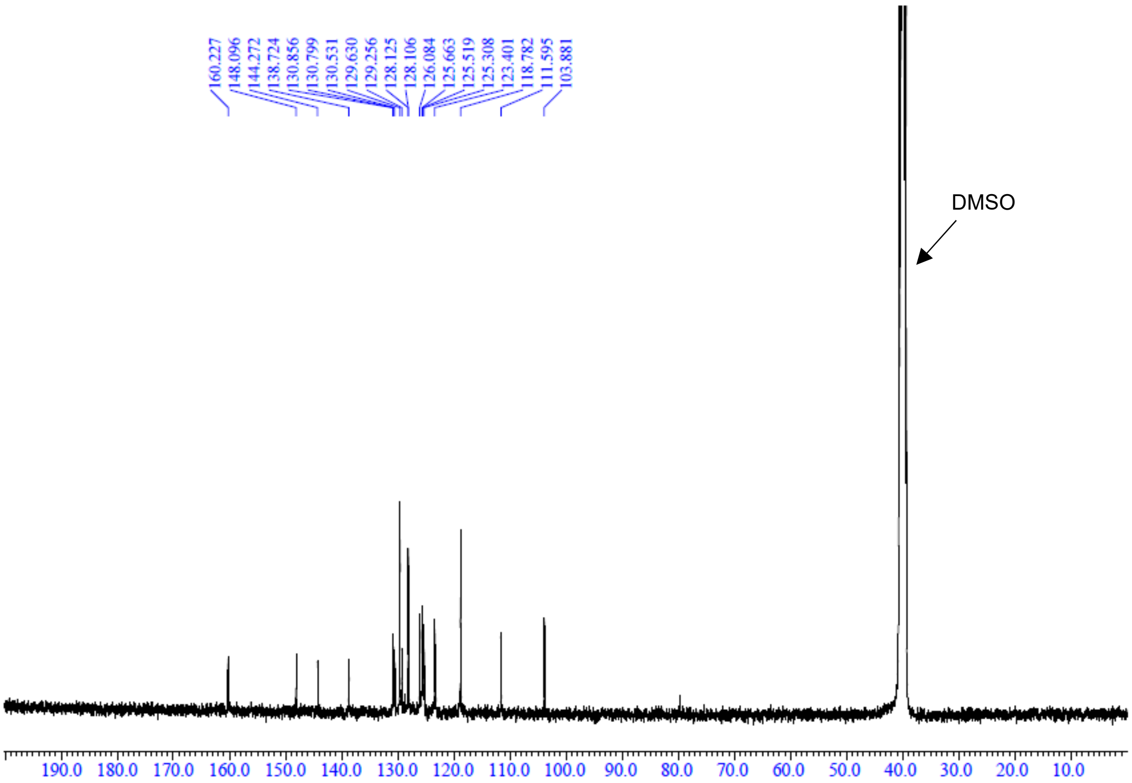

^{1}H NMR of **11**

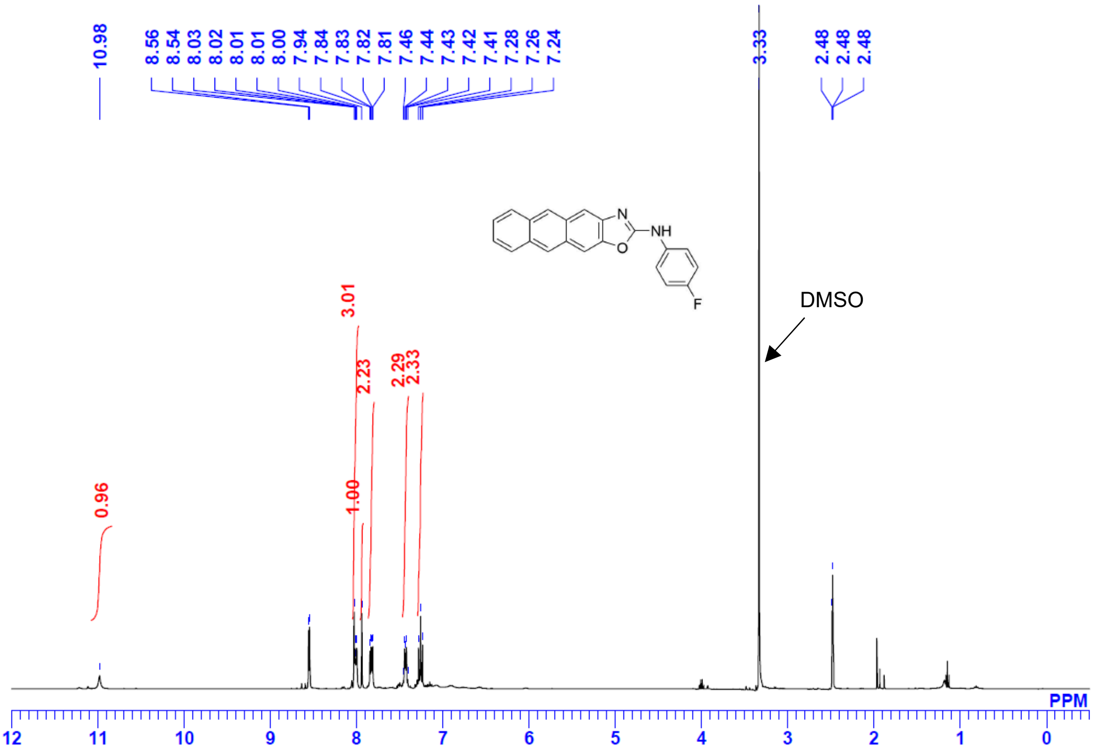

^{13}C NMR of **11**

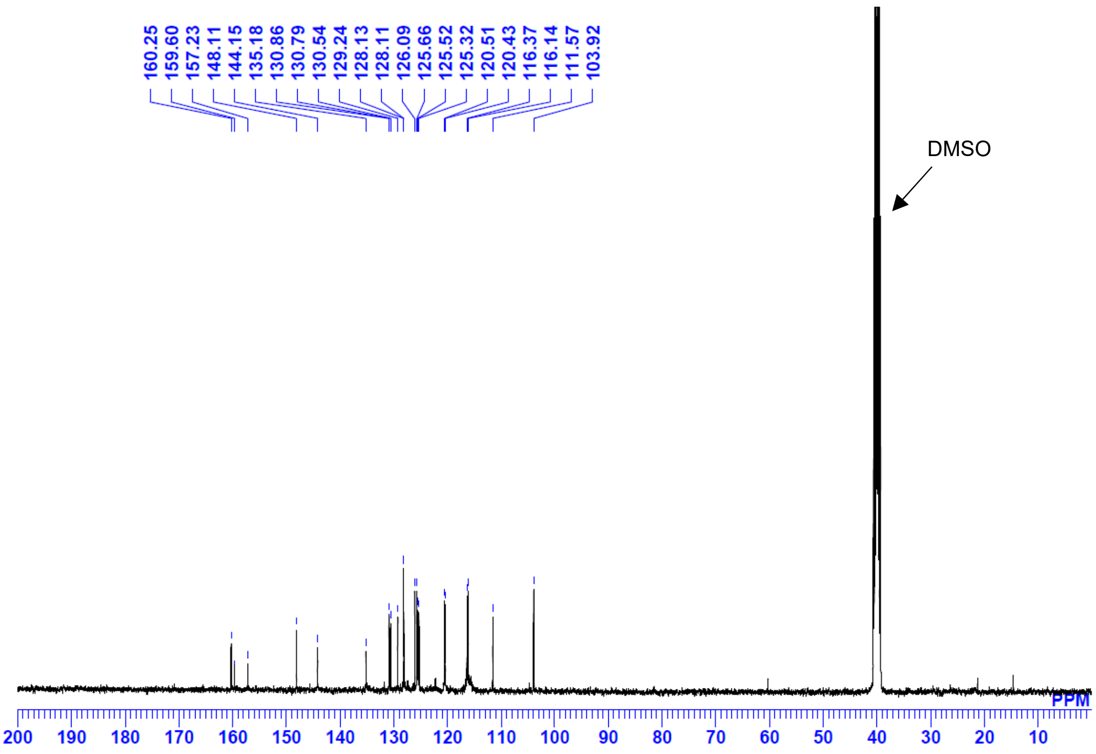

^{19}F NMR of **11**

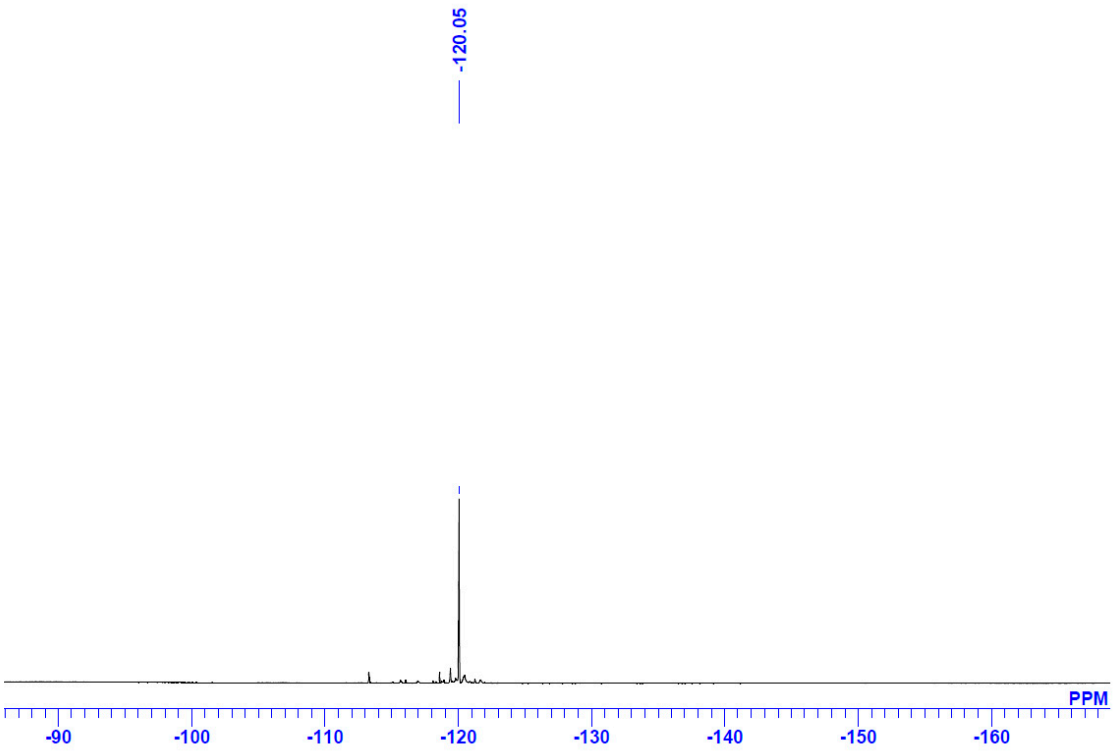

^{1}H NMR of **12**

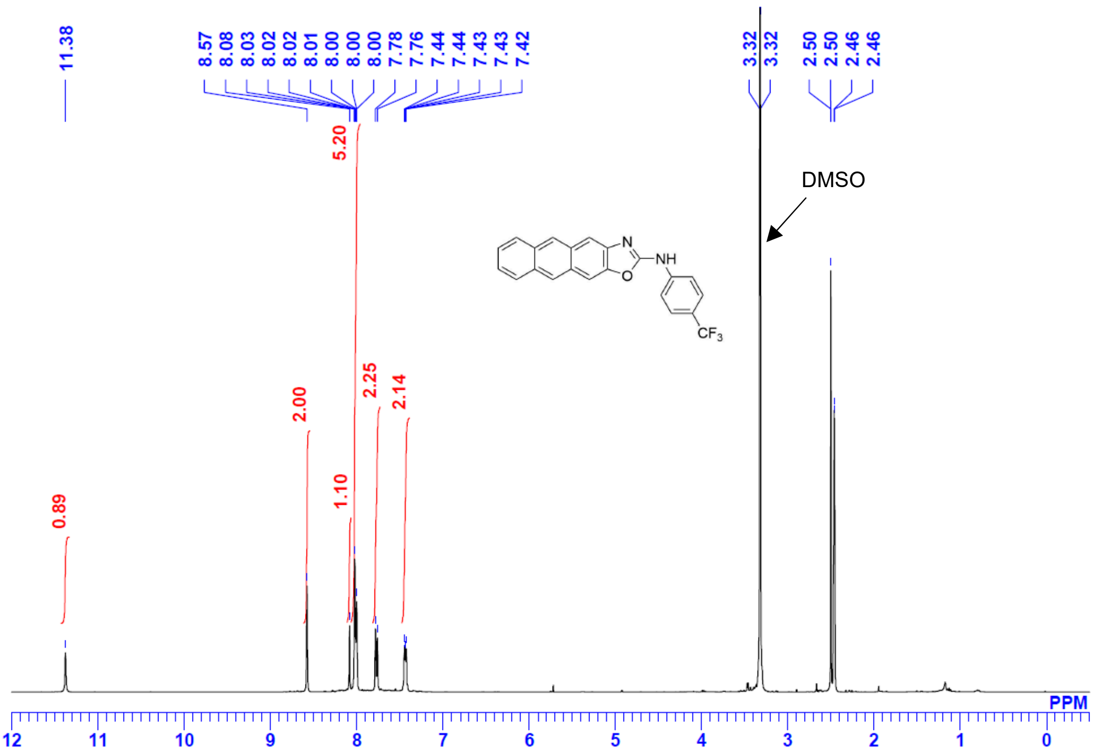

^{13}C NMR of **12**

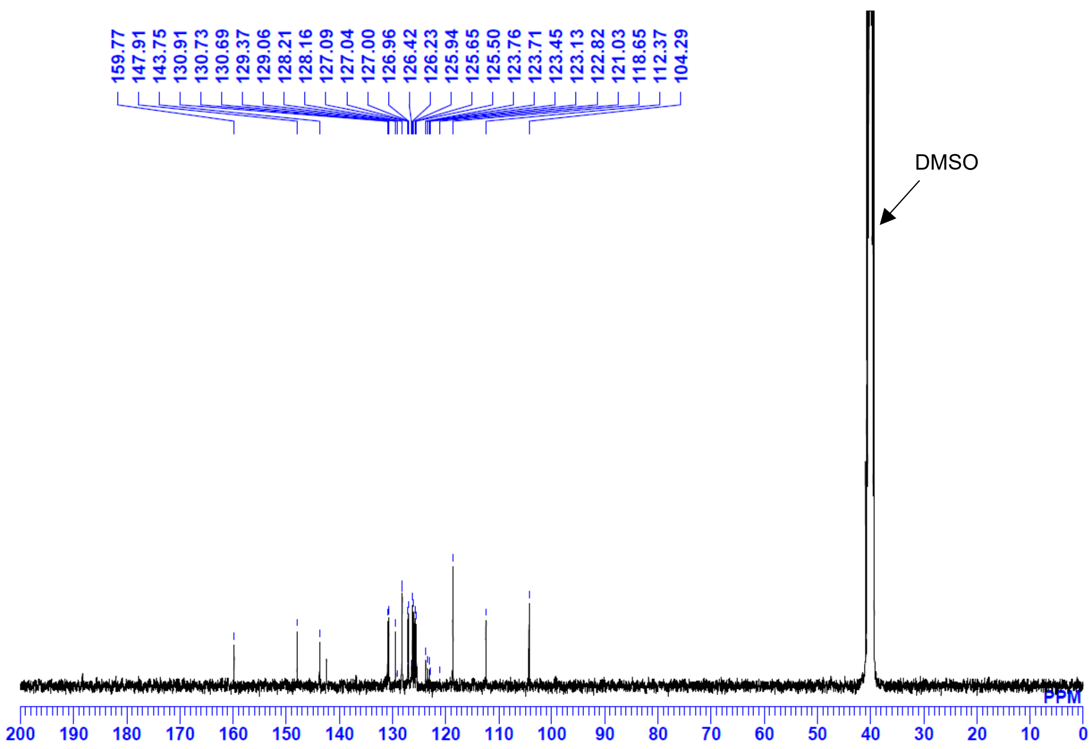

^{19}F NMR of **12**

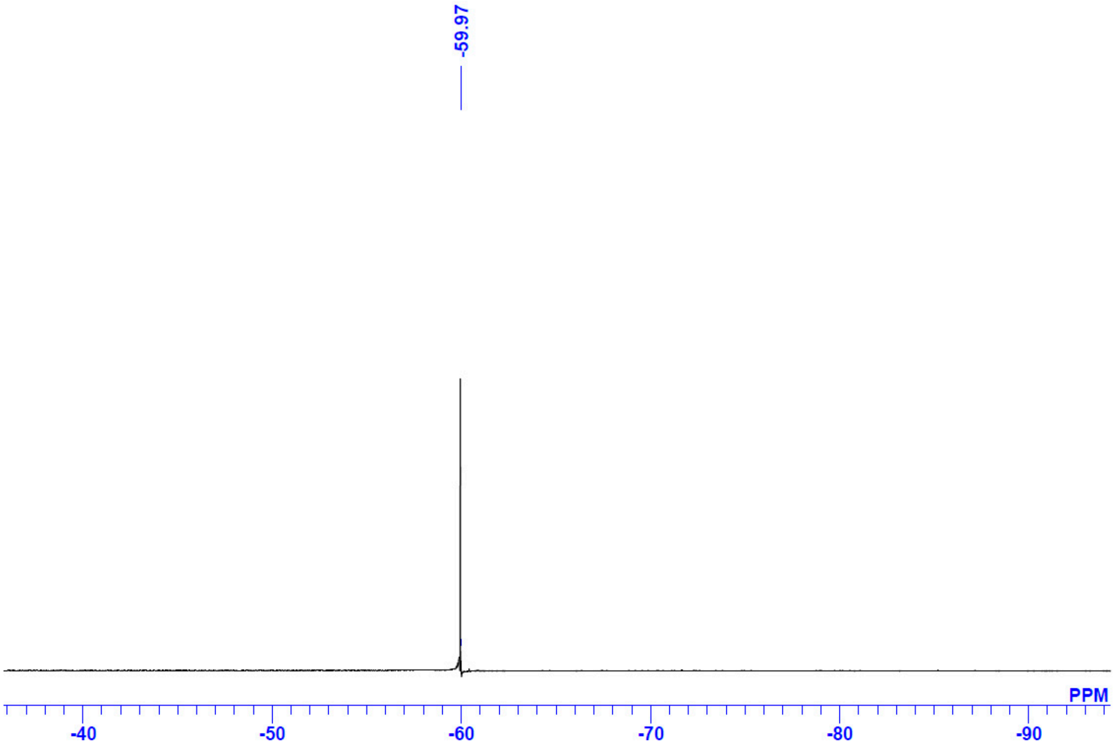

Supplement: Supplementary file 1 [file molecules-30-00319-s001.zip › molecules-3416325-supplementary.pdf]
